# Supplementary material for: Who’s afraid of the big bad boar? Assessing the effect of wild boar presence on the occurrence and activity patterns of other mammals
Source: PLoS One. 2020 Jul 14;15(7):e0235312. doi: 10.1371/journal.pone.0235312 (PMC7360034; doi:10.1371/journal.pone.0235312)
Supplement: Attachment — Submitted filename: PONE-D-19-25832_reviewer.pdf [file pone.0235312.s002.pdf]

PLOS ONE

# Who’s afraid of the big bad boar? Assessing the effect of wild boar presence on the occurrence and activity patterns of other mammals

--Manuscript Draft--

| | |
|---|---|
| **Manuscript Number:** | PONE-D-19-25832 |
| **Article Type:** | Research Article |
| **Full Title:** | Who’s afraid of the big bad boar? Assessing the effect of wild boar presence on the occurrence and activity patterns of other mammals |
| **Short Title:** | Who’s afraid of the big bad boar? Assessing the effect of wild boar over other mammals |
| **Corresponding Author:** | Carlos Benhur Kasper, Dr.<br>Universidade Federal do Pampa - Campus Sao Gabriel<br>Sao Gabriel, Rio Grande do Sul BRAZIL |
| **Keywords:** | invasive species; mammals; Neotropics; Atlantic Forest; Activity Patterns |
| **Abstract:** | Wild boar are considered one the world’s worst invasive species and linked to biodiversity loss, competition for resources, predation of native species, and habitat modifications. In this study, we use camera traps to evaluate whether the invasive wild boar had an effect on the medium-sized mammal community of a protected area in southern Brazil. Based on photographic records, we evaluated whether the presence and relative abundance of wild boar was associated with a decrease in diversity or change in activity of medium-sized mammals. All comparisons were made between samples where wild boar were present or absent. The records of each camera during a season were considered a sample. The wild boar was the fourth most common species in the study area being present in 7.8% of the photographic records. The species richness of mammals was not negatively affected by the occurrence of wild boar and most common species did not exhibit changes in the daily activity pattern. However, we recorded an increase in the time elapsed between an observation of wild boar and the record of the next species relative to the average latency period observed among other mammalian species. This average latency period was similar to that observed in the case of large predators such as Puma, and its increase could be reflective partly of the avoidance of native species to wild boar. Nevertheless, our results show that the effect of invasive wild boar on the mammal community is not large as expected. |
| **Order of Authors:** | Êmila Silveira de Oliveira |
| | Manoel Ludwig da Fontoura Rodrigues |
| | Magnus Machado Severo |
| | Tiago Gomes dos Santos |
| | Carlos Benhur Kasper, Dr. |
| **Additional Information:** | |
| **Question** | **Response** |
| **Financial Disclosure**<br><br>Enter a financial disclosure statement that describes the sources of funding for the work included in this submission. Review the submission guidelines for detailed requirements. View published research articles from *PLOS ONE* for specific examples.<br><br>This statement is required for submission | CAPES (Comissão de Aperfeiçoamento de Pessoal de Nível Superior) Brasil. Scholarship grant to ESO. The funders had no role in study design, data collection and analysis, decision to publish, or preparation of the manuscript. |

*Powered by Editorial Manager® and ProduXion Manager® from Aries Systems Corporation*

| | |
|---|---|
| and **will appear in the published article** if the submission is accepted. Please make sure it is accurate.<br><br>**Unfunded studies**<br>Enter: *The author(s) received no specific funding for this work.*<br><br>**Funded studies**<br>Enter a statement with the following details:<br>• Initials of the authors who received each award<br>• Grant numbers awarded to each author<br>• The full name of each funder<br>• URL of each funder website<br>• Did the sponsors or funders play any role in the study design, data collection and analysis, decision to publish, or preparation of the manuscript?<br>• **NO** - Include this sentence at the end of your statement: *The funders had no role in study design, data collection and analysis, decision to publish, or preparation of the manuscript.*<br>• **YES** - Specify the role(s) played.<br><br>* typeset | |
| **Competing Interests**<br><br>Use the instructions below to enter a competing interest statement for this submission. On behalf of all authors, disclose any competing interests that could be perceived to bias this work—acknowledging all financial support and any other relevant financial or non-financial competing interests.<br><br>This statement **will appear in the published article** if the submission is accepted. Please make sure it is accurate. View published research articles from *PLOS ONE* for specific examples. | NO authors have competing interests |

| | |
|---|---|
| **NO authors have competing interests**<br><br>Enter: *The authors have declared that no competing interests exist.*<br><br>**Authors with competing interests**<br><br>Enter competing interest details beginning with this statement:<br><br>*I have read the journal's policy and the authors of this manuscript have the following competing interests: [insert competing interests here]*<br><br>* typeset | |
| **Ethics Statement**<br><br>Enter an ethics statement for this submission. This statement is required if the study involved:<br><br>• Human participants<br>• Human specimens or tissue<br>• Vertebrate animals or cephalopods<br>• Vertebrate embryos or tissues<br>• Field research<br><br>Write "N/A" if the submission does not require an ethics statement.<br><br>General guidance is provided below. Consult the submission guidelines for detailed instructions. **Make sure that all information entered here is included in the Methods section of the manuscript.** | The study was conducted according the licences SISBIO / ICMBio nº 47667-1 and 59075-1. |

Powered by Editorial Manager® and ProduXion Manager® from Aries Systems Corporation

| | |
|---|---|
| **Format for specific study types**<br><br>**Human Subject Research (involving human participants and/or tissue)**<br>• Give the name of the institutional review board or ethics committee that approved the study<br>• Include the approval number and/or a statement indicating approval of this research<br>• Indicate the form of consent obtained (written/oral) or the reason that consent was not obtained (e.g. the data were analyzed anonymously)<br><br>**Animal Research (involving vertebrate animals, embryos or tissues)**<br>• Provide the name of the Institutional Animal Care and Use Committee (IACUC) or other relevant ethics board that reviewed the study protocol, and indicate whether they approved this research or granted a formal waiver of ethical approval<br>• Include an approval number if one was obtained<br>• If the study involved *non-human primates*, add *additional details* about animal welfare and steps taken to ameliorate suffering<br>• If anesthesia, euthanasia, or any kind of animal sacrifice is part of the study, include briefly which substances and/or methods were applied<br><br>**Field Research**<br><br>Include the following details if this study involves the collection of plant, animal, or other materials from a natural setting:<br>• Field permit number<br>• Name of the institution or relevant body that granted permission | |
| **Data Availability**<br><br>Authors are required to make all data underlying the findings described fully available, without restriction, and from the time of publication. PLOS allows rare exceptions to address legal and ethical concerns. See the PLOS Data Policy and FAQ for detailed information. | Yes - all data are fully available without restriction |

| | |
|---|---|
| A Data Availability Statement describing where the data can be found is required at submission. Your answers to this question constitute the Data Availability Statement and **will be published in the article**, if accepted.<br><br>**Important:** Stating 'data available on request from the author' is not sufficient. If your data are only available upon request, select 'No' for the first question and explain your exceptional situation in the text box.<br><br>Do the authors confirm that all data underlying the findings described in their manuscript are fully available without restriction? | |
| **Describe where the data may be found in full sentences. If you are copying our sample text, replace any instances of XXX with the appropriate details.**<br><br>• If the data are **held or will be held in a public repository**, include URLs, accession numbers or DOIs. If this information will only be available after acceptance, indicate this by ticking the box below. For example: *All XXX files are available from the XXX database (accession number(s) XXX, XXX.*).<br>• If the data are all contained **within the manuscript and/or Supporting Information files**, enter the following: *All relevant data are within the manuscript and its Supporting Information files.*<br>• If neither of these applies but you are able to provide **details of access elsewhere**, with or without limitations, please do so. For example:<br><br>*Data cannot be shared publicly because of [XXX]. Data are available from the XXX Institutional Data Access / Ethics Committee (contact via XXX) for researchers who meet the criteria for access to confidential data.*<br><br>*The data underlying the results presented in the study are available from (include the name of the third party* | All relevant data are within the manuscript and its Supporting Information files. |

| | |
|---|---|
| *and contact information or URL).*<br>• This text is appropriate if the data are owned by a third party and authors do not have permission to share the data.<br><br>* typeset | |
| Additional data availability information: | Tick here if the URLs/accession numbers/DOIs will be available only after acceptance of the manuscript for publication so that we can ensure their inclusion before publication. |

Powered by Editorial Manager® and ProduXion Manager® from Aries Systems Corporation

Manuscript
Click here to access/download;Manuscript;Main Manuscript _ Who is afraid of Big Bad Boar_Plos One.docx

# Who's afraid of the big bad boar? Assessing the effect of wild boar presence on the occurrence and activity patterns of other mammals

Êmila Silveira de Oliveira¶1, Manoel Ludwig da Fontoura Rodrigues¶2, Magnus Machado Severo¶3, Tiago Gomes dos Santos¶4 and Carlos Benhur Kasper¶*5

1 - Laboratório de Biologia de Mamíferos e Aves (LABIMAVE) e Programa de Pós Graduação em Ciências Biológicas, Universidade Federal do Pampa (UNIPAMPA), São Gabriel. Rua Aluízio Barros Macedo, Br 290 km 423, Bairro Piraí, São Gabriel, Brazil.

2 - Programa de Pós Graduação em Biologia Animal, Universidade Federal do Rio Grande do Sul (UFRGS). Departamento de Zoologia, Prédio 43435, Instituto de Biociências, Universidade Federal do Rio Grande do Sul, Av. Bento Gonçalves 9500, Porto Alegre/RS, Brazil. CEP: 91540-000.

3 - Instituto Chico Mendes de Conservação da Biodiversidade - Ministério do Meio Ambiente (ICMBio / MMA). Praça Pref. Luis Martins, 30 - Mostardas/RS, Brazil. CEP: 96.207-000.

4 - Laboratório de Estudos em Biodiversidade Pampiana (LEBIP), Universidade Federal do Pampa (UNIPAMPA), São Gabriel, Brazil. Rua Aluízio Barros Macedo, Br 290 km 423, Bairro Piraí, São Gabriel, Brazil.

5 - Laboratório de Biologia de Mamíferos e Aves (LABIMAVE) e Programa de Pós Graduação em Ciências Biológicas, Universidade Federal do Pampa (UNIPAMPA), São Gabriel. Rua Aluízio Barros Macedo, Br 290 km 423, Bairro Piraí, São Gabriel, Brazil.

* Corresponding author: E-mail: cbkasper@yahoo.com.br

¶ These authors contributed equally to this work

**Abstract:** Wild boar are considered one the world's worst invasive species and linked to biodiversity loss, competition for resources, predation of native species, and habitat modifications. In this study, we use camera traps to evaluate whether the invasive wild boar had an effect on the medium-sized mammal community of a protected area in southern Brazil. Based on photographic records, we evaluated whether the presence and relative abundance of wild boar was associated with a decrease in diversity or change in activity of medium-sized mammals. All comparisons were made between samples where wild boar were present or absent. The records of each camera during a season were considered a sample. The wild boar was the fourth most common species in the study area being present in 7.8% of the photographic records. The species richness of mammals was not negatively affected by the occurrence of wild boar and most common species did not exhibit changes in the daily activity pattern. However, we recorded an increase in the time elapsed between an observation of wild boar and the record of the next species relative to the average latency period observed among other mammalian species. This average latency period was similar to that observed in the case of large predators such as Puma, and its increase could be reflective partly of the avoidance of native species to wild boar. Nevertheless, our results show that the effect of invasive wild boar on the mammal community is not large as expected.

**INTRODUCTION**

Biological diversity faces numerous threats globally, including the effect caused by invasive species, which is often irreversible in many cases [1]. After habitat loss, the invasion of alien species is viewed as the second biggest threat to biodiversity [2]. Invasive species tend to show rapid adaptation to new environments and can affect the ecology of native species due to predation, competitive exclusion, and niche drive causing local extinctions in some cases [3].

The wild boar (*Sus scrofa*) is one the top 100 worst invasive species in the world [2]. The original geographical distribution of wild boar encompasses almost all Eurasia, making this one of the most widely distributed mammal species [4]. Currently the wild boar can be found on all continents except for Antarctica [5]. First records in Brazil date from late 1980’s and early 1990’s [6], with the species now being found in all ecoregions except for Amazonia, which is an invasion speed of 149.6 $km^2$ year [7].

The invasive potential of the wild boar may be related to hybridization with domestic pigs [8]. In tropical countries, hybrid specimens of up to 350 kg have been recorded [9]. In south eastern Brazil, wild adult males have a body mass of 100 to 130 kg but can reach 200 kg or more [10]. Moreover, the large supply of food and the mild climate contribute to more than one parturition event a year, with four to twelve piglets per farrowing [1]. These factors are associated with the virtual absence (or low abundance) of natural predators, allowing exotic wild boar to achieve high densities.

In several countries in the invasive range, different forms of the feral swine have been linked to the decline of biodiversity. Here we will consider this invasive form of feral swine, *S. scrofa*, as wild boa[illegible]he effect on animal communities has been observed in invertebrates, amphibians, reptiles, birds and mammals and is related to competition for resources, predation, habitat use and destruction of nests [4]. In addition to these alarming factors, wild boar can be

a reservoir of many viral, bacterial, and parasitic diseases that can be transmitted to native fauna, domestic animals, and humans [1, 11, 12, 13, 14, 15].

Although wild boar are found throughout the majority of Brazil, Uruguay, and Argentina, information on its effect on biodiversity is limited. Recently in southern Brazil, it was confirmed that these animals decrease the probability of detection and occupation of mammals, negatively affecting the occurrence of some species [16]. Changes in the activities of native pigs due to niche overlap with the wild boar have also been observed in the Atlantic Forest and in the Pantanal [10, 17]. Another study carried out in southeast Brazil found that wild boar alters the physical structure of streams, causing silting in these watercourses [18]. The effect of wild boar on degradation of forest vegetation has also been observed [19]. Such effects may cause a decrease in local diversity; however, only few studies have assessed the effects of wild boar on species or groups of animals.

The effects of wild boar on native animals, especially mammals, are still poorly understood. Could the wild boar be affecting the community of medium- and large mammals in preserved environments? Our study aims to evaluate the possible effects of the presence of wild boar in a preserved environment, verifying whether their presence changes the richness and diversity of mammals or is related to possible changes in their activity patterns. Our hypothesis is that the presence of the wild boar would alter composition and change the behaviour of other mammals. Thus, we predict that (1) presence of wild boar will inhibit the occurrence of certain species, lowering richness and diversity indices of native mammals where the wild boar is present or more active, and (2) the pattern of mammalian activity will differ where wild boar are more active.

## MATERIALS AND METHODS

**Study area:** The study was conducted in the National Forest (FLONA) of São Francisco de Paula, located in the region of Campos de Cima da Serra, in the northeast of the state of Rio Grande do Sul, coordinates 29°25′S, 50°23′O. The climate of this region is classified as temperate; mild summer and rigorous winter, with severe and frequent frosts (Cfb) [20]. The average annual temperature is 10.5°C (50.9°F). The FLONA of São Francisco de Paula is a Conservation Unit of sustainable use that has an area of 1,606 hectares (ha[illegible] is formed mainly by Mixed Ombrophilous Forest (900 ha of native forest), with reforestations of Brazilian pine *Araucaria angustifolia* (Bertol.) Kuntze (390 ha), *Pinus* sp. (229 ha) and small cultivations of *Eucalyptus* sp. (34 ha) [21]. The FLONA of São Francisco de Paula is considered a region of "highest priority" for conservation in Brazil according to the Mapping of Priority Areas for the Conservation of the Atlantic Forest [21].

**Data collection:** Data collection occurred in five periods of three months: winter (July to September) and spring (October to December) of 2016 and, autumn (April to June), winter (July to September) and spring (October to December) of 2017.

During each period, five photographic traps (Bushnell model Aggressor) were installed at five points located on trails and roads inside FLONA. These points were located at an average distance of approximately 1.7 km (ranging from 1.5 to 1.9 km) from each othe[illegible]his distribution of cameras was set to cover different sectors of the Conservation Unit. Traps were configured to operate 24 hours a day, with a minimum interval of 30 minutes after each record. Due to this time of interval, all records obtained by each camera were considered as independent. No lures or baits were used.

**Data analysis:** Initially, we described the occurrence of medium- and large mammals in the National Forest to understand the context of the environment where the wild boar are invading. Data on the number of records were used as an index of relative abundance of each species. This index is used to analyse the possible effects of wild boar in our study area, as described below. The photographic records of wild boar in FLONA was used to derive ecological data, including information about relative abundance, the proportion of males, females and young and the activity period.

We considered the records of each sampling point (camera trap) on each field trip as a sample unit. As we have five sites sampled at five time points, this corresponds to 25 sample units. Each sample unit was classified by the presence and absence of wild boar in the corresponding period. Next, we compared diversity estimates of native mammals in samples with and without wild boar by using a non-asymptotic approach based on rarefaction (interpolation) and extrapolation curves. Comparisons were made by inspection of curves for a standardized sample size (i.e. mammal abundance) and for a standardized sample coverage (a measure of sample completeness) [22]. For each type of curve, we plotted confidence intervals (95% based on 50 re-samples by bootstrapping), corresponding to three orders of Hill numbers [23]: q = 0 (species richness), q = 1 (the exponential of Shannon's entropy index), and q = 2 (the inverse of Simpson's concentration index). The analysis was based on species abundance data, available from the software iNEXT online [24]. We also use Spearman's coefficient of correlation as an approach to estimate the effect of wild boar abundance on the abundance of other mammal species. These analyses of correlation were made only including species with 10 or more records.

In order to verify possible temporal responses of mammal species to the presence of wild boar, we calculated the average time elapsed between records of native species as well as the time elapsed until the next species is seen after recording a wild boar. The same procedure

was adopted for the puma (*Puma concolor*), which is the top predator in this environment. We compared three groups of time periods (considering each camera individually): 1) time elapsed after a record of wild boar and the next mammal species; 2) time elapsed after a record of a puma and the next mammal species; and 3) time between records of two consecutive species (excluding wild boar and puma). To compare the periods between records, a Kruskal–Wallis test was applied, and a comparison of the groups was verified using the Mann–Whitney *U* test. Statistical analyses were performed using PAST software version 2.16 [25] and R [26].

The daily activity of mammals was analysed and described through circular statistical analysis [27]. Using this method we estimated: a) the mean angle (μ), which represents the direction, i.e. the mean time of day during which each species was active; b) the length of the mean vector (r), a measure of data concentration around the daily cycles, ranging from 0 (scattered data) to 1 (concentrated data on the same direction), and; c) the circular standard deviation (SD) and confidence intervals (95% and 99%) for μ. We summarized the major results of daily activity using rose diagrams. Records of all common species (more than 10 records) were separated by taxon, and between samples with or without records of wild boar.

The Rayleigh's Uniformity Test was used to calculate the probability of a null hypothesis that the data are uniformly distributed around the circadian cycles ($P>0.05$) [27]. Thus, a significant result of the Rayleigh test ($P<0.05$) is associated with longer mean vector and larger Z values, indicating that the data are not uniformly distributed [28]; or there is a concentration of mammal's activity during circadian cycles. Changes in the activity period of mammals in response of the presence of wild boar were evaluated by comparing the patterns of daily activity exhibited in sampled sites without records of wild boar. Thus, we used the Watson–Williams F-test [27], a non-parametric test that compares the lengths of the mean vectors among samples. If the null hypothesis is rejected ($P<0.05$), this indicates the mammal species present changes in patterns of daily activity between these conditions. To compare

activity patterns, we used only species that were present in more than 10 records in both conditions (with and without wild boar), as a smaller number of records could affect the statistical power. All circular analyses were performed in ORIANA 2.02 [28]. For the Watson–Williams F-test, we selected to incorporate a correction factor, based on the concentration, to correct for possible bias.

## RESULTS

**Diversity and patterns of circannual activity:** With a sampling effort of 1191 night traps, 644 photographic records of 21 medium and large mammal species were obtained, including the wild boar (Table 1). The species with the highest number of records were *Cerdocyon thous* (20.2% of the records), *Dasyprocta azarae* (18.2%), and *Leopardus guttulus* (17.7%). The wild boar was the fourth most common species, with 7.8% of the photographic records.

**Table 1:** Photographic records of medium and large mammal species in São Francisco de Paula National Forest, Rio Grande do Sul state, Brazil.

| **Taxon** | **Sampling points** | | | | | **Total** |
|---|---|---|---|---|---|---|
| | **A** | **B** | **C** | **D** | **E** | |
| Didelphimorphia | | | | | | |
| *Didelphis albiventris* | 0 | 1 | 0 | 0 | 0 | **1** |
| Pilosa | | | | | | |
| *Tamandua tetradactyla* | 1 | 0 | 0 | 0 | 2 | **3** |
| Cingulata | | | | | | |
| *Dasypus novemcinctus* | 0 | 0 | 0 | 1 | 1 | **2** |
| *Dasypus* sp. | 0 | 0 | 0 | 0 | 1 | **1** |
| Artiodactyla | | | | | | |
| *Mazama gouazoubira* | 27 | 3 | 3 | 0 | 10 | **43** |
| *Mazama nana* | 0 | 0 | 1 | 0 | 0 | **1** |
| *Pecari tajacu* | 0 | 4 | 2 | 0 | 0 | **6** |

| | | | | | | |
|---|---|---|---|---|---|---|
| *Sus scrofa* * | 7 | 11 | 1 | 3 | 28 | **50** |
| Carnivora | | | | | | |
| *Cerdocyon thous* | 79 | 1 | 11 | 11 | 28 | **130** |
| *Lycalopex gymnocercus* | 0 | 0 | 0 | 0 | 1 | **1** |
| *Leopardus pardalis* | 4 | 6 | 1 | 0 | 16 | **27** |
| *Leopardus guttulus* | 30 | 26 | 11 | 23 | 24 | **114** |
| *Leopardus wiedii* | 4 | 7 | 5 | 0 | 3 | **19** |
| *Leopardus* sp. | 4 | 1 | 3 | 3 | 8 | **19** |
| *Puma concolor* | 26 | 3 | 6 | 3 | 5 | **43** |
| *Puma yagouaroundi* | 0 | 1 | 0 | 0 | 1 | **2** |
| *Lontra longicaudis* | 0 | 0 | 0 | 1 | 0 | **1** |
| *Eira barbara* | 0 | 1 | 0 | 0 | 0 | **1** |
| *Galictis cuja* | 0 | 1 | 1 | 1 | 2 | **5** |
| *Nasua nasua* | 0 | 8 | 0 | 2 | 0 | **10** |
| *Procyon cancrivorus* | 17 | 0 | 1 | 5 | 3 | **26** |
| Rodentia | | | | | | |
| *Cuniculus paca* | 0 | 22 | 0 | 0 | 0 | **22** |
| *Dasyprocta azarae* | 0 | 48 | 1 | 9 | 59 | **117** |
| | **199** | **144** | **47** | **62** | **192** | **644** |

The wild boar were recorded over all sampling periods and were more frequent in the winter months in both years (Figure 1). Solitary males represent 70% of records of this species. Females with farrow accounted for a further 8% of the records, including a video with three females accompanied by 14 farrow. Attempts to identify individuals resulted in the identification of twenty-six individuals of *S. scrofa*. Due to the low recapture rate, it was not possible to estimate wild boar density in the sampled area. Half of the identified individuals occur at the same sample point and almost all (22 individuals) were recorded for only one of three month period. Only three individuals (two females with farrow and one solitary male) were recorded across two months and only one individual (a solitary male) was recorded for three months. None of the identified wild boar was recorded at different sampling points.

**Figure 1:** Number of *Sus scrofa* photographic records along five field sampling periods in São Francisco de Paula National Forest, Southern Brazil.

When considering the sampling periods (lasting three months) and each sampling point as independent samples, we noted the presence of the wild boar in 60% of the samples. We recorded that samples with and without the occurrence of wild boar do not differ in diversity of medium-sized mammals for order q = 0 (i.e. species richness), as the confidence intervals of both sample-size-based and sample completeness curves overlapped (Figure 2A). However, species diversity for orders q > 0 tended to be higher in samples with the occurrence of wild boar (Figure 2B and 2C). In addition, we did not find any significant negative correlation between the relative abundance of wild boar and the relative abundance of most common species (with more than ten photographic records) (Table 2). The unique significant positive correlation was found between two species: *Leopardus guttulus* and *Dasyprocta azarae*.

**Figure 2:** Comparison of diversity estimates for medium-sized mammals recorded in the samples with and without wild boar in São Francisco de Paula National Forest, Southern Brazil. Sample-size-based (left) and sample completeness (right) curves for Hill numbers of order q = 0, q = 1, and q = 2. The 95% confidence intervals were obtained by bootstrapping (based on 50 replicates).

**Table 2:** Correlation between the number of records of wild boar and the number of records of the most common species (species with more than 10 records) in São Francisco de Paula National Forest, Southern Brazil

| **Species** | **Rho** | **p-value** |
|---|---|---|
| Artiodactyla | | |
| *Mazama gouazoubira* | 0.37 | 0.06 |
| Carnivora | | |
| *Cerdocyon thous* | 0.36 | 0.06 |
| *Puma concolor* | 0.13 | 0.51 |
| *Leopardus pardalis* | 0.20 | 0.32 |
| *Leopardus wiedii* | 0.22 | 0.28 |
| *Leopardus guttulus* | 0.43 | 0.02 |

| | | |
|---|---|---|
| *Nasua nasua* | 0.10 | 0.61 |
| *Procyon cancrivorus* | 0.01 | 0.96 |
| Rodentia | | |
| *Cuniculus paca* | 0.28 | 0.17 |
| *Dasyprocta azarae* | 0.44 | 0.02 |

**Patterns of daily activity:** The average latency period between observing a wild boar and the record of the next species was 53:58 hours. This period was very similar to the latency period observed for the Puma (53:39 hours) and differs from that observed amongst other mammalian species, which averaged 41:55 hours. A comparison of latency periods among species showed no difference between the three groups (H=5.15; p=0.075). Paired analysis indicates a significant difference only in the latency "after Puma" versus the latency "among other species" (z=2.13; p=0.032).

The circular statistical analysis showed that the activity of most mammal species was not uniformly distributed around the daily cycle (Rayleigh test) (Table 3). The highest concentration in daily activity was recorded for *Cerdocyon thous* and *Procyon cancrivorus* (Table 3). Only *Mazama gouazoubira* (in sites without records of wild boar) and *Sus scrofa* presented pattern of activity uniformly distributed around the daily cycle. The wild boar were active during day and night time with peaks of activity at sunrise and first hour of sunset (Figure 3). Females were more diurnal and crepuscular and farrow were recorded exclusively at daytime.

**Table 3:** Summary of circular statistical analysis testing for uniformity in daily activity of mammal species monitored in São Francisco de Paula National Forest, Southern Brazil. Abbreviations: rc = record condition: sites with records of wild boar (+), sites without records of wild boar (-), n = number of observations, μ = mean angle (in time of day), r = mean vector, Circular SD = circular standard deviation, 95% CI = upper and lower confidence intervals of 95% for μ, 99% CI = upper and lower confidence intervals of 99% for μ, Rayleigh Test = Z values and probabilities associated: *(P<0.05), **(P<0.01).

| Species | rc | n | μ | r | Circular SD | 95% CI | 99% CI | Rayleigh Test |
|---|---|---|---|---|---|---|---|---|
| *Mazama gouazoubira* | - | 24 | 13:21 | 0.16 | 110.23° | 06:31-20:11 | 04:22-22:20 | 0.59 |
| | + | 21 | 23:14 | 0.50 | 67.07° | 21:06-01:22 | 20:26-02:03 | 5.33** |
| *Cerdocyon thous* | - | 68 | 23:39 | 0.60 | 57.83° | 22:42-00:37 | 22:24-00:54 | 24.55** |
| | + | 67 | 22:35 | 0.65 | 53.44° | 21:43-23:27 | 21:27-23:43 | 28.07** |
| *Leopardus guttulus* | - | 35 | 02:48 | 0.32 | 86.21° | 00:06-05:30 | 23:15-06:21 | 3.64* |
| | + | 82 | 02:19 | 0.23 | 98.16° | 23:48-04:49 | 23:01-05:36 | 4.35* |
| *Puma concolor* | - | 22 | 20:11 | 0.38 | 79.48° | 17:20-23:01 | 16:27-23:55 | 3.21* |
| | + | 23 | 01:42 | 0.46 | 70.92° | 23:28-03:57 | 22:46-04:39 | 4.97** |
| *Procyon cancrivorus* | - | 13 | 23:16 | 0.72 | 46.75° | 21:22-01:11 | 20:46-01:47 | 6.68** |
| | + | 13 | 00:37 | 0.74 | 44.47° | 22:49-02:26 | 22:15-03:00 | 7.12** |
| *Dasyprocta azarae* | - | 12 | 14:39 | 0.55 | 62.68° | 11:59-17:19 | 11:08-18:09 | 3.63* |
| | + | 106 | 12:54 | 0.55 | 62.48° | 12:03-13:45 | 11:47-14:01 | 32.28** |
| *Sus scrofa* | | 47 | 00:02 | 0.08 | 130.09° | 13:53-10:11 | 10:42-13:22 | 0.27 |

**Figure 3:** Activity pattern of *Sus scrofa* in São Francisco de Paula National Forest, Southern Brazil

Most of the common species do not exhibit changes in the daily activity pattern when comparing sites were the wild boar was and was not recorded (Table 4). Only *M. gouazoubira* and *P. concolor* changed the daily activity pattern, since the activity of these two species presented higher concentration (i.e. higher mean vector values) in the sites with records of wild boar (Figure 4 and 5).

**Table 4:** Summary of Watson–Wheeler Test comparing daily activity pattern of six most common mammal species in the presence and absence of wild boar, in São Francisco de Paula National Forest, Southern Brazil.

| Species | W |
|---|---|
| *Mazama gouazoubira* | 7.79 * |
| *Cerdocyon thous* | 2.64 |
| *Leopardus guttulus* | 0.32 |
| *Puma concolor* | 7.52* |
| *Procyon cancrivorus* | 2.39 |
| *Dasyprocta azarae* | 2.29 |

**Figure 4:** Activity pattern of grey brocket deer (*Mazama gouazoubira*) in samples without presence of wild boar (left) and in the presence of wild boar (right).

**Figure 5:** Activity pattern of puma (*Puma concolor*) in samples without presence of wild boar (left) and in the presence of wild boar (right).

## DISCUSSION

The National Forrest of São Francisco de Paula is of great importance for biodiversity conservation. We recorded 21 species of medium and large mammals including several threatened taxa in southern Brazil [29], such as *Tamandua tetradactyla*, *Leopardus guttulus, L. pardalis, L. wiedii*, *Puma yagouaroundi*, *P. concolor*, *Eira barbara*, *Lontra longicaudis*, *Nasua nasua*, *Cuniculus paca, Dasyprocta azarae*, *Mazama gouazoubira* and *M. nana*. Other medium-sized mammals previously recorded at this Conservation Unit have arboreal habitats, such as the Southern Brown Howler Monkey (*Alouatta guariba*), or are typical of the open areas that surround this National Forest and are recorded only occasionally such as the Pampas Deer (*Ozotoceros bezoarticus*), Maned Wolf (*Chrysocyon brachyurus*) or the hog nosed skunk (*Conepatus chinga*) [30, 31]. The first records of wild boar in this National Forest occurred in April 2007. In the same year, an attempt was made to control the species, without success, due to their difficulty of capture [32]. Currently, the wild boar appears well-established and adapted to the region as it has made use of the National Forest across all seasons.

The higher frequency of wild boar recorded at the National Forest in the winter months may be influenced by the availability of araucaria seeds in this period. Araucaria seeds are part

of the wild boar diet throughout its distribution in the Mixed Ombrophilous Fores[illegible]0, 19, 33]. The wild boar may affect the regeneration of araucarias trees, considered "critically endangered" by the IUCN [34] not only by the predation of seeds but the extensive disturbance caused when they churn the soi[illegible]3]. This activity, apart from affecting the regeneration of the species, may also influence the ecology of mammals that also consume these seeds, especially small rodents [35]. Focused studies are needed to evaluate the effect of wild boar on the population dynamics of the araucaria trees as it was beyond the scope of this study.

The influence of wild boar on the assemblage of medium-sized mammals is difficult to detect and is poorly studied. In our study, samples where wild boar were present exhibited higher diversity of medium-sized mammal orders (q>0), but presented similar diversity estimates for species richness with samples without wild boar (q=0[illegible]he last result is contrary to those obtained by Hegel [16] where samples with the presence of wild boar presented lower mammalian richness. However, our results are congruent with Pantanal, were the wild boar has not been shown to be a direct threat to the native fauna. In this environment, wild boar and other species showed signs of niche division due to morphological and behavioural differences, allowing its coexistence with native pigs, even in periods with low availability of resources [36, 37, 38]. We do not believe that wild boar positively affect the environment as the species is still using the same microhabitats selected by other species. In this case, the effect of wild boar can be more severe, as it is occupying preferred habitats of a larger variety of animals. However, our results showed no direct negative relationship between the species recorded and the wild boa[illegible]n samples with records of wild boar, we record a decrease of dominance of certain species, such *Cerdocyon thous* that are the most common species in the samples without boar. The correlation of the boar with *Dasyprocta azarae* (the most common species to co-occur with wild boar) may reflect the use of the same environment. Both agouti and wild boar tend to use more forested sample sites when araucaria seeds are more abundant and represent an important

resource for the agouti (35, 39]. The correlation of the wild boar with *L. guttulus* may result from a casual consequence of using the same environments. We hypothesize that the wild boar uses environments with high availability of araucaria seeds (fact not evaluated) that allow high abundances of small rodents [35, 40, 41], that, in turn, might attract small cats

The wild boar showed a cathemeral pattern of activity. The lack of preference for a certain period of the day, but with peaks of activity at the beginning and the end of the day, are characteristics already expected for the species [42]. The activity distributed throughout the day may indicate a low anthropic effect in the studied area, as wild boar tend to modify their behaviour, altering their use during a certain period in response to anthropic pressure [43]. Another possible influence investigated here were changes in activity pattern of mammals in response to different intensity or frequency of wild boar. The analyses were conducted only with species frequently recorded in samples with and without wild boar. Only two species exhibited some temporal response to the presence of wild boar: the grey brocket deer (*M. gouazoubira*) and the puma (*P. concolor*). The grey brocket deer presented cathemeral activity in samples where wild boar was not recorded and show activity more concentrated at night, especially close to midnight, in samples with the presence of wild boar (Figure 4). Puma that presented peaks in activity in the first hours of night and day in samples with no records of wild boar change to peaks of activity close to midnight (Figure 5). This is interesting as the wild boar exhibits two peaks of activity: near sunset and near sunrise (figure 3). These changes in the activity of grey brocket deer and puma could reflect some avoidance of wild boa[illegible]

Although we did not find major shifts in activity pattern of most mammals associated to the presence of the boar, we did observe a delay in the time of recording other mammal species following a record of wild boar. The time gap before other record after the passage of a wild boar was similar to the time elapsed after the passage of the puma (*P. concolor*). Thus, it is likely the presence of the wild boar has an inhibiting effect on the use of the environment

similar to that of a large predator. Therefore, the wild boar could affect the population dynamics of other species (even temporarily) in the use of the habitat.

Biological communities with lower species richness tend to become more prone to biological invasions as they do not offer "biological resistance" against exotic species [44]. Areas with a diverse community of mammals may be more resistant to wild boar invasion due to exploratory competition of other mammals diminishing the invader's resources [45]. In this way, the lack of evidence of the effect of wild boar on the richness and diversity of recorded mammals may suggest that the conservation and high biodiversity of the study area are temporarily ‘protecting’ native mammal species from the effect. However, it is important to keep in mind that this greater diversity may reduce, but not necessarily prevent, the invasive capacity of the wild boar [45], further reinforcing the importance of the conservation of natural environments and consequently, of native biodiversity. Finally, it is important to understand that effects of invasive species can begin with small modifications in the behaviour of native species, and it is difficult to evaluate what effect this will have on the biological dynamics of the environment.

## ACKNOWLEDGEMENTS

We thank to São Francisco de Paula National Forest administration for the logistic support, especially to Edenice Brandão Avila de Souza.

**REFERENCES**


1- Lombardi R, Geymonat G, Berrini R. El jabalí en el Uruguay: Problema, desafio y oportunidad. Montevideo: Florestal Atlántico Sur; 2015.

2- Lowe S, Browne M, Boudjelas S. 100 of the world's most invasive species: a selection from the global invasive species database. ISSG, Auckland. Invasive Species Specialist Group (ISSG), World Conservation Union; 2000.

3- Mooney HA, Cleland EE. The evolutionary impact of invasive species. Proc Natl Acad Sci USA. 2001; 98:5446-5451.

4- Massei G, Genov PV. The environmental impact of wild boar. Galemys. 2004; 16:135-145.

5- Long JL. Introduced mammals of the world: their history distribution and influence. Collingwood: CSIRO; 2003.

6- Pereira-Neto AO, Riet-Correa F, Méndez MDC. Javali: um predador a ser evitado no Rio Grande do Sul. In: Schild AL, Riet-Correa F, Méndez MDC, Ferreira JLM, editors. Laboratório Regional de Diagnóstico: Doenças diagnosticadas no ano de 1991. Pelotas: Editora Universitária; 1992. pp 42–48.

7- Salvador CH (2012) Ecologia e manejo de javali (*Sus scrofa* L.) na América do Sul. Ph.D. Thesis, Universidade Federal do Rio de Janeiro. 2012. Available from: https://www.researchgate.net/publication/282659323_Ecologia_e_manejo_de_javali_Sus_scr

ofa_L_na_America_do_Sul_Ecology_and_management_of_Eurasian_wild_boar_Sus_scrofa_L_in_South_America

8- García G, Vergara J, Lombardi R. Genetic characterization and phylogeography of the wild boar *Sus scrofa* introduced into Uruguay. Genet Mol Biol. 2011; 34:329-337.

9- Canevari M, Vaccaro O. Guía de mamíferos del sur de América del Sur. Buenos Aires: LOLA; 2007.

10- Salvador CH, Fernandez FAZ. Using the Eurasian wild boar phenotype as a 97 basis to document a new process of invasion by *Sus scrofa* L. in a Neotropical biodiversity hotspot. Wildl Biol Pract. 2014; 10:22-29.

11- Rosell C, Fernández-Llario P, Herrero YJ. El jabalí (*Sus scrofa* LINNAEUS, 1758). Galemys. 2001; 13:1-25.

12- Gortázar C, Ferroglio E, Höfle U, Frölich K, Vicente J. Diseases shared between wildlife and livestock: a European perspective. Eur J Wildl Res. 2007; 53:241–256.

13- Meng XJ, Lindsay DS, Sriranganathan N. Wild boars as sources for infectious diseases in livestock and humans. Philos Trans R Soc Lond B Biol Sci. 2009; 364: 2697-2707

14- Mendina-Filho LH, Wallau MO, Reis TX. O javali no Pampa: Contexto, biologia e manejo. Santana do Livramento: Sindicato Rural de Santana do Livramento; 2015.

15- Silva V, Trevisol IM, Kramer B, Bordin LC, Nones J, Dambrós R, Salvador CH, Tortato MA. Monitoramento sorológico de Peste Suína Clássica em suídeos asselvajados (*Sus scrofa*) no estado de Santa Catarina. Brasília: O Biológico; 2015.

16- Hegel CGZ. Padrões de ocupação do javali (*Sus scrofa* L.) na Mata Atlântica sul brasileira. M.Sc. Thesis. Universidade Regional Integrada do Alto Uruguai e das Missões. 2017. Available from: http://www.uricer.edu.br/cursos/arq_trabalhos_usuario/3414.pdf

17- Galetti M, Camargo H, Siqueira T, Keuroghlian A, Donatti CI, Jorge MLSP et al. Diet Overlap and Foraging Activity between Feral Pigs and Native Peccaries in the Pantanal. PLoS ONE. 2015 Nov 4. doi: 10.1371/journal.pone.0141459.

18- Rosa CA. Mamíferos Exóticos Invasores no Brasil: situação atual, riscos potenciais e impactos da invasão de porcos selvagens em Florestas Tropicais. Ph.D. Thesis, Universidade Federal de Lavras. 2016. Available from: http://repositorio.ufla.br/jspui/handle/1/10799

19- Hegel CGZ, Marini MA. Impact of the wild boar, *Sus scrofa*, on a fragment of Brazilian Atlantic Forest. Neotrop Biol Conserv. 2013; 8:17-24.

20- Köppen W. Climatologia: con un estúdio de los climas de la tierra. Cidad de México: Fundo de Cultura Econômica; 1948.

21- ICMBio. Floresta Nacional São Francisco de Paula/RS: Educação ambiental na Floresta Nacional de São Francisco de Paula/RS. In: Revisão do Plano de manejo, equipe FLONA SFP/RS. 2007 [cited 10 September 2017]. Available from: http://www.florestanacional.com.br/flonasaochico.html

22- Chao A, Jost L. Coverage-based rarefaction and extrapolation: standardizing samples by completeness rather than size. Ecology. 2012; 93:2533-2547

23- Chao A, Gotelli NJ, Hsieh TC, Sander EL, Ma KH, Colwell RK, Ellison AM. Rarefaction and extrapolation with Hill numbers: a framework for sampling and estimation in species diversity studies. Ecol Monogr. 2014; 84:45-67.

24- Chao A, Ma KH, Hsieh TC. iNEXT (iNterpolation andEXTrapolation) online: software for interpolation and extrapolation of species diversity. 2016 [cited 20 August 2018]. Available from: http://chao.stat.nthu.edu.tw/wordpress/software_download.

25- Hammer O, Harper DAT, Ryan PD. PAST: Paleontological Statistic software package for education and data analysis. Paleontol Electron. 2001; 4:1-9.

26- R Core Team. R: A language and environment for statistical computing. 2016 [cited 4 April 2018]. Available from: https://www.R-project.org.

27- Zar JH. Biostatistical Analysis. 4th ed. Upper Saddle River: Prentice Hall; 1999.

28- Kovach WL. Oriana – Circular Statistics for Windows [online]. 2011. [cited 18 September 2018]. Available from: https://www.kovcomp.co.uk/oriana/oriana4man.pdf.

29- FZB. Lista das espécies da fauna gaúcha ameaçadas de extinção. 2014 [cited 12 June 2018]. Available from: http://www.fzb.rs.gov.br/upload/2014090911580809_09_2014_especies_ ameacadas.pdf.

30- Marques RV, Cademartori CV, Pacheco SM. Mastofauna no Planalto das Araucárias, Rio Grande do Sul, Brasil. Rev Bras Biocienc. 2011; 9:278-288.

31- Marques RV, Fabian ME. The manned wolf in the ecotone between Forest and grasslands at the limits of its distribution in a subtropical environment. Biosci J. 2013; 29:751-759.

32- Boziki DM, Printes EC, Souza EBA, Soligo AJ. Diagnóstico do javali “*Sus scrofa*” na Floresta Nacional de São Francisco de Paula e em sua zona de amortecimento. In: Hernandez ACV, Duarte MM, Reinehr R. Educação para a Sustentabilidade. São Francisco de Paula: UERGS; 2017. pp 304-326.

33- Batista GO. O javali (*Sus scrofa* Linnaeus, 1758) na região do Parque Nacional das Araucárias: percepções humanas e sua relação com regeneração de *Araucaria angustifolia* (Bertol.) Kuntze. M.Sc. Thesis, Universidade Federal de Santa Catarina. 2015. Available from: https://repositorio.ufsc.br/xmlui/handle/123456789/156542

34- Thomas P. *Araucaria angustifolia*. In: The IUCN Red List of Threatened Species. 2013 [28 January 2017]. Available from: www.iucnredlist.org.

35- Iob G, Vieira EM. Seed Predation of *Araucaria angustifolia* (Araucariaceae) in the Brazilian Araucaria Forest: Influence of Deposition Site and Comparative Role of Small and 'Large' Mammals. Plant Ecol. 2008; 198:185-196.

36- Sicuro, FL, Oliveira, LF. Coexistence of Peccaries and Feral Hogs in the Brazilian Pantanal Wetland: An Ecomorphological View. J Mammal. 2002; 83:207-217.

37- Desbiez ALG, Santos SA, Keuroghlian A, Bodmer RE. Niche partitioning between sympatric populations of native white-lipped peccary (*Tayassu pecari*), collared peccary (*Tayassu tajacu*). J Mammal. 2009; 90:119-128.

38- Oliveira-Santos LGR, Dorazio RM, Tomas WM, Mourão G, Fernandez FAZ. No evidence of interference competition among the invasive feral pig and two native peccary species in a Neotropical wetland. J Trop Ecol. 2011; 27:557-561.

39- Ribeiro JF, Vieira EM. Interactions between a seed-eating neotropical rodent, the Azara's agouti (*Dasyprocta azarae*), and the Brazilian 'pine' *Araucaria angustifolia*. Austral Ecol. 2014; 39:279-287.

40- Vieira EM, Ribeiro JF, Iob G. Seed predation of *Araucaria angustifolia* Araucariaceae) by small rodents in two areas with contrasting seed densities in the Brazilian Araucaria forest. J Nat Hist. 2011; 45:843-854.

41- Grazzini G, Mochi-Júnior CM, Oliveira H, Pontes JS, Gatto-Almeida F, Tiepolo LM. Identidade, riqueza e abundância de pequenos mamíferos (Rodentia e Didelphimorphia) de área de Floresta com Araucária no estado do Paraná, Brasil. Pap Avulsos Zool. 2015; 55:217-230

42- Puertas FH. A invasão do javali na Serra da Mantiqueira: Aspectos populacionais, uso do habitat e sua relação com o Homem. M.Sc. Thesis, Universidade Federal de Lavras. 2015. Available from: http://repositorio.ufla.br/bitstream/1/9779/1/DISSERTACAO_A%20invas%C3%A3o%20do%20javali%20na%20serra%20da%20mantiqueira%20Aspectos%20populacionais%2C%20uso%20do%20habitat%20e%20sua%20rela%C3%A7%C3%A3o%20com%20o%20Homem.pdf

43- Podgórski T, Bas G, Jędrzejewska B, Sönnichsen L, Śnieżko S, Jędrzejewski W, Okarma H. Spatiotemporal behavioral plasticity of wild boar (*Sus scrofa)* under contrasting conditions of human pressure: primeval forest and metropolitan area. J Mammal. 2013; 94:109-119

44- Elton CS. The ecology of invasions by animals and plants. London: Springer Science Business Media; 1958.

45- Garza SJ, Tabak MA, Miller RS, Farnsworth ML, Burdett C. Abiotic and biotic influences on home-range size of wild pigs (*Sus scrofa*). J Mammal. 2018; 99:97-107.

## SUPPORTING INFORMATION

S1: Datasheet with activity patterns of wild boar and other mammals in São Francisco de Paula National Forest, Rio Grande do Sul state, Brazil, and comparative statistics

Figure 1

Click here to access/download;Figure;Figure 1.tif

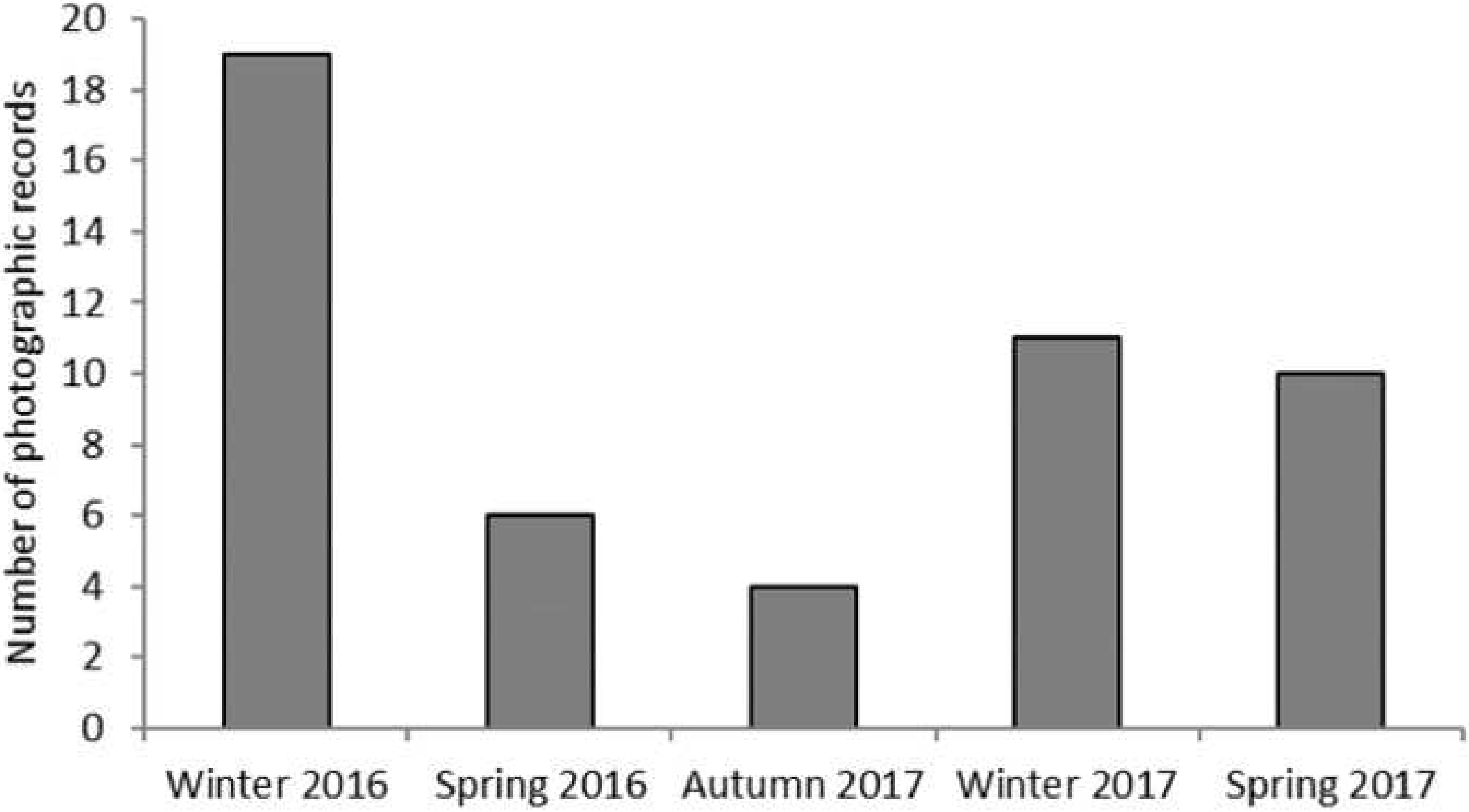

Figure 2

Click here to access/download;Figure;Figure 2.tif

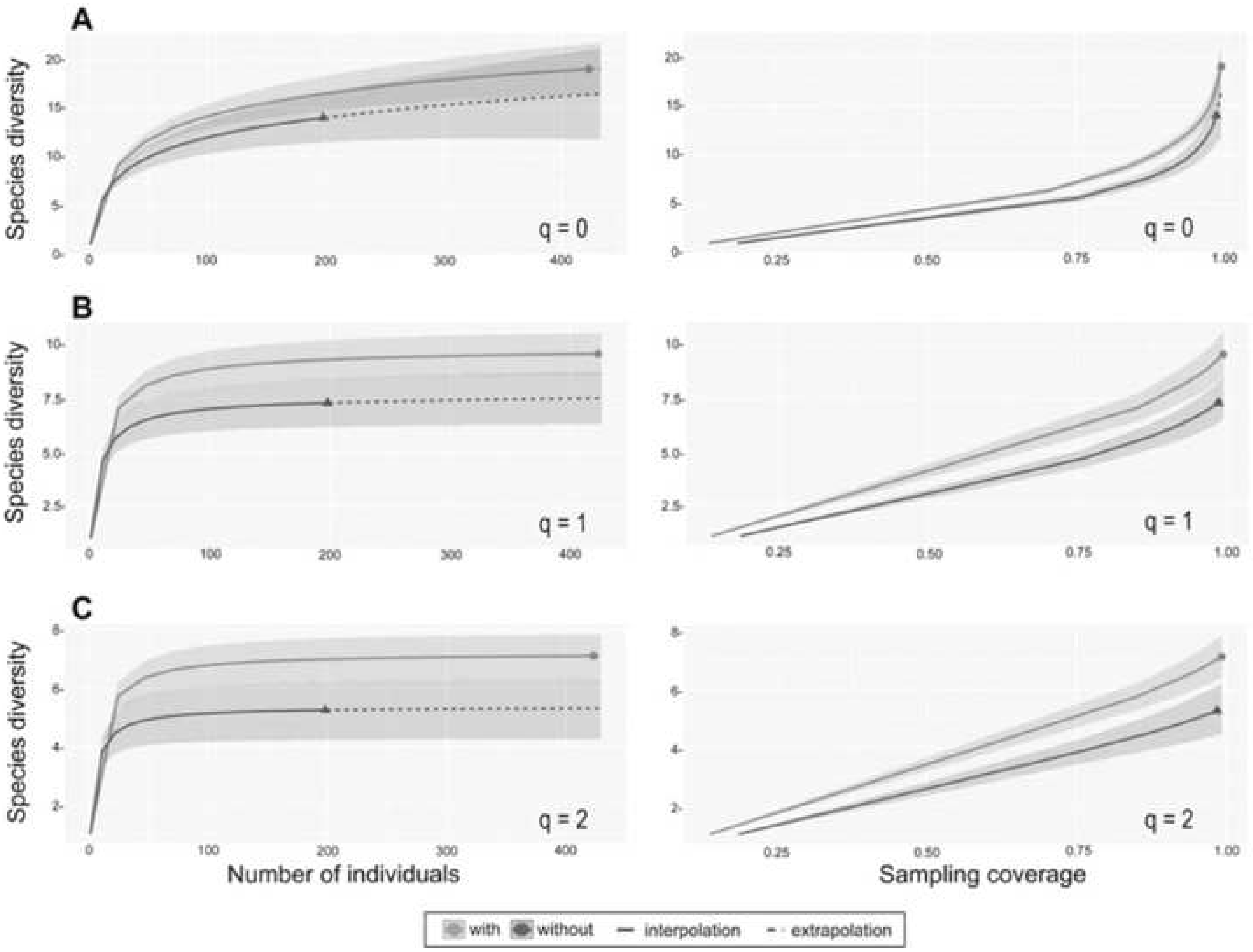

Figure 3 Click here to access/download;Figure;Figure 3.tif

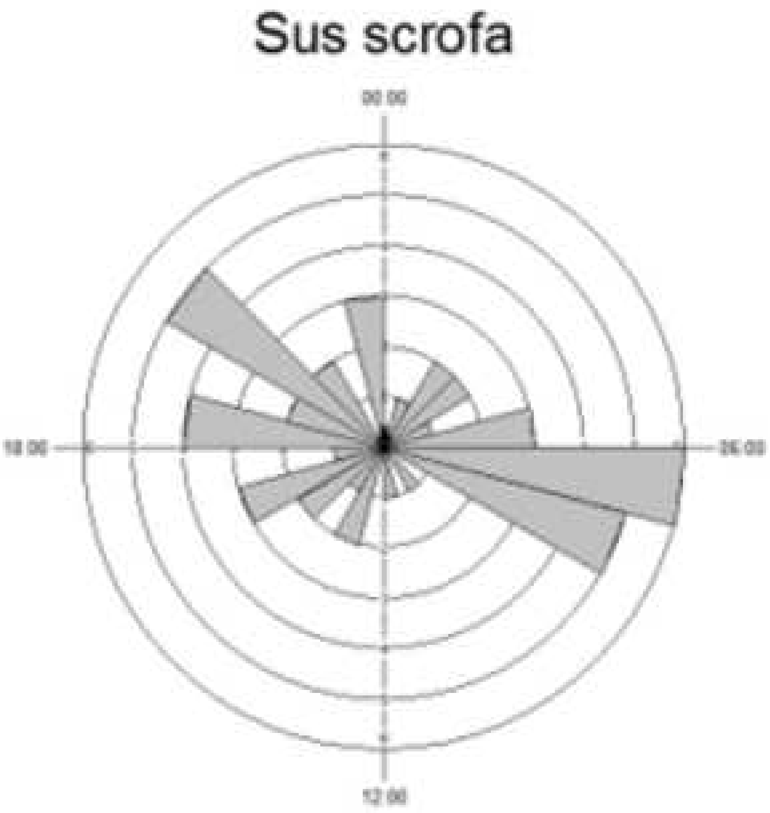

Figure 4

Click here to access/download;Figure;Figure 4.tif

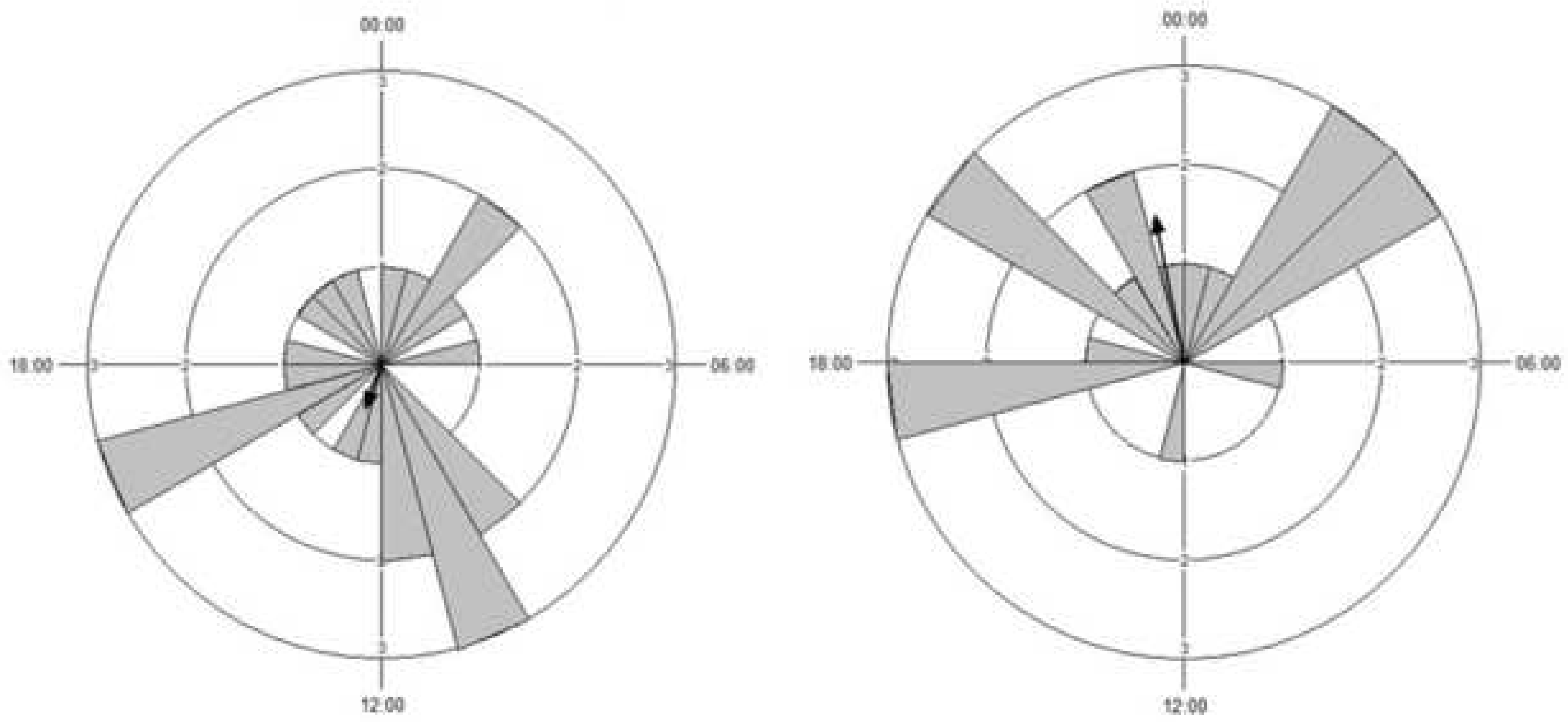

Figure 5

Click here to access/download;Figure;Figure 5.tif

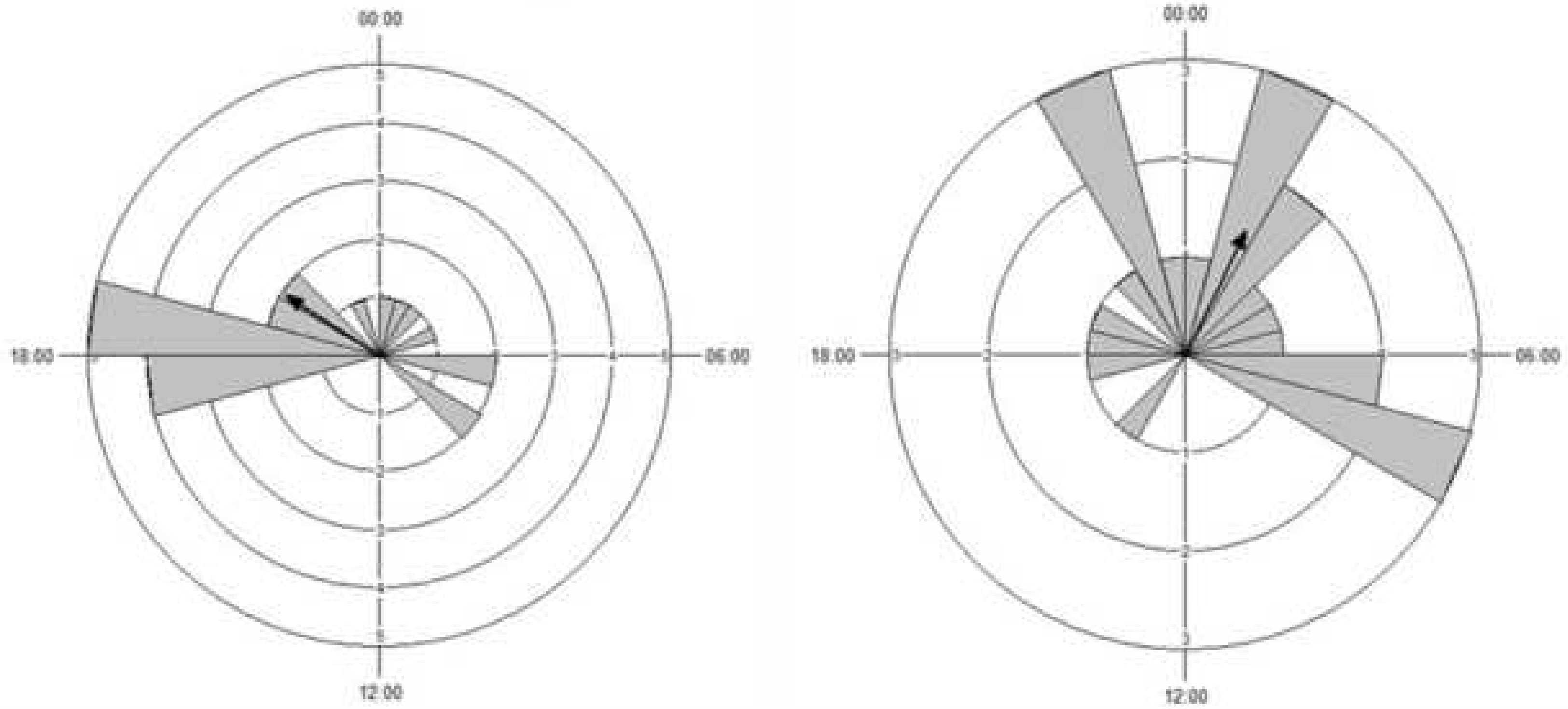

Click here to access/download
**Supporting Information**
S1_Dataset activity pattern.xlsx
