# Supplementary material for: Who’s afraid of the big bad boar? Assessing the effect of wild boar presence on the occurrence and activity patterns of other mammals
Source: PLoS One. 2020 Jul 14;15(7):e0235312. doi: 10.1371/journal.pone.0235312 (PMC7360034; doi:10.1371/journal.pone.0235312)
Supplement: Attachment — Submitted filename: Rebuttal letter_Plos One_revisada.docx [file pone.0235312.s003.docx]

**Rebuttal Letter _ Manuscript PONE-D-19-25832 _ entitled:**

**“Who’s afraid of the big bad boar? Assessing the effect of wild boar presence on the occurrence and activity patterns of other mammals”**

Below, we respond to each of the reviewer comments for the manuscript.

Line 68: So do you have ibryds between wild boar and domesstic pigs? If yes say it explicitely in the texy and do not confound wild boar with feral swine.

**Response:** We have rewritten this to make it clearer.

Line 77: Can you mention explicitely the species you mean here?

**Response:**  We have now added the species name to the text.

Line 91: “One problem I see here is that you are not taking into account the time elapsend since wild boar (or feral pigs) established themselves in the study area. Presence per se may mean very different things if it refers to newly colonised areas or areas with a long lasting stable presence. Moreover the abundance of wild boar can again be deciding in determining its impact on animal population.”

**Response:** The information regarding the establishment of the wild boar in this region was addressed in the discussion section. We included this information in the study area section to improve understanding. We evaluated the effect of abundance indirectly based on the number of records of wild boar, which we considered equivalent to the intensity of use.

Line 92: So here you have to evaluate activity patterns shift with wild boar of / and activity patterns shift within the same species in areas with and without wild boar.

**Response:** The entire study area was used by wild boar, but not at the same time. Therefore, we evaluated activity pattern shifts when wild boar were present in each specific sector (sampled by the camera trap unit), compared to the areas where wild boar were temporarily not recorded. We think that is clearer in the manuscript, but we can improve this section if necessary.

Line 104: This size seems to me a bit small to discriminate areas with and without wild boar, I do not know the home range size in Suoth America but in Southern Europe it is on avereage of 300 ha, as consequence an area of 1600 ha where wild boar is present is compeltely occupied by this species and the actual presence of individuals is determined by micro environmental characteristics rather than actual presence or absence

**Response:** The reviewer is absolutely correct. Due to these factors, our comparisons were based on the temporal use of parts of the study area relative to other areas. Equivalent areas (with similar characteristics) were not available in the absence of wild boar. Therefore, we evaluated the immediate impact of the temporal presence of wild boar on other species. This approach was possible because the wild boar were not present in all areas of the National Forest at all times. Rather, they were using some sectors during a given period, and other sectors during other periods (as shown in our results). Thus, we evaluated the effect on other mammals when wild boar were present during a period, despite their presence throughout the region over time.

Line 116: Some description of sampling sites are needed to understand how different they were. The presence or absence of species could have been caused by a number of causes and not only by wild boar presence. The best option could be that the enviromental factors were all equal among the five points so wild boar presence could be considered a reasonable cause of diversity. However, this has to be shown by the Authors if this is the case.

**Response:** A basic description has been added to this section.

Line 122: Only description or did you quantified mammals presence?

**Response:** Beyond an estimation of the number of wild boar recorded, we did not quantify the number of individuals. Rather, we used the number of recordings as an index of the relative abundance of each species.

Line 145: I think this can be seen also in the perspective of wild boar, that is an obvious prey for puma, so a complex web of interactions may be established and this can not be simply decribed with a simple univariate approach

**Response:** We would suggest caution regarding the assumption that wild boars are invariably prey of puma. In Brazil, puma (*Puma concolor*) are smaller than individuals from North America and the Patagonia region, weighing roughly 40 kg (22-70 [Paglia et al. 2012]). By contrast, wild boar usually weigh more than 100 kg. In fact, puma are known to eat smaller prey compared to other big cats. In the book Neotropical Cats, Tadeu G. Oliveira compiled data on the diet of puma in different countries and indicated that MWVP for the puma varies from 3.7 to 32.4 kg. Jaguars present an MWVP of 113.2 kg in some regions of Brazil for comparison. While we agree that puma indeed could prey on young wild boar, we doubt this is a regular occurrence.. Nevertheless, we made a revision regarding the diet of puma in South America and found some references to the consumption of wild boar, though usually at low rates. We refer to this data in the discussion section.

Line 193 and 197: “These data suggest that wild boar establishment in the area was quite initial and caraterized by low densities. I t would be very useful to understand the meaning of the whole paper to have more clear information on the timing of wild boar establishment in the area and any further indication of the population size and distribution. As said in the comments to the aims of the paper you can found in the introduction theses information could be very relevant to the interpretation of the results” “Again this gives the impression of a relatively recent establishment of the popuation accompanied by a low density”

**Response:** In the study area section we have added information about the time of invasion by the wild boar and included a reference about the relevance of the problem of this exotic species for the Conservation Unit. They have been well established for many years, but we have scarce documentation about this. Personal communication with employees of the CU reports numbers around ‘200 wild boars killed in last few years’. Unfortunately, we do not have official data about this nor explicit collaboration with the managers of the CU regarding this information.

Line 205: Where the sampling points with and without wild boar different from the environmental point of view? Could the presence of wild boar be seen as the outcome of particular conditions? And, more important, was this the case for the other species? I imagine that if the sampling areas were different the mammal communities were also different but this could not be attibuted to wild boar presence but to the different environmental situation. As an example from Europe if you look for polecats (a medium size mustelid carnivore) you can have by far more opportunities to find them close to small rivers and creeks rather than in arid environments.

**Response:** We acknowledge this concern. However, as we have explained in previous comments, there are two points to be considered: 1^st^: wild boar were recorded in all points except one sampling period (there were five periods of three months for each sample point). Therefore, we compared the effect of the temporal presence of wild boar in each section. We used the terms “with” and “without” wild boar in a more temporal rather than spatial context. We considered a sample point to be without wild boar if we did not record the species within 3 months. However, in a broader perspective they were present. As a result, our analysis used temporal windows and looked for immediate responses from the other mammals. 2^nd^: we did not access microhabitat information to correlate with the mammal community, and this was not our intent. We determined that there were no profound differences between sample points, resulting in differences in the medium-sized mammal community.

Comment on Table 2:

Positive or negative? As they are they seems to me positive possibly meaning similar ecological needs or more casual local abundance of the two species

**Response:** We included the value of the correlation, but considered a nonsignificant correlation to be irrelevant. We discussed significant correlations in the discussion section.

Line 231: What about the latency between wild boar and puma?

**Response:** We included this latency period and the reverse (wild boar after puma). Specifically, the latency was more than 9 days between the record of a puma after the record of wild boar! However, this was not directly comparable. Our comparison included “wild boar versus a group (any mammal)”, “puma versus a group (any mammal)” and “group versus any group (any mammal versus any mammal)”. A latency period of puma versus wild boar would be a more specific comparison (a specific species versus another specific species). We thank the reviewer for this pertinent and interesting question.

Comment on Table 4: Could this mean an active search of wild boar by pumas ?

**Response:** New analyses (comment 231) would indicate that this is definitely not the case, given a latency period of almost 9 days between wild boar and puma records in our data.

Line 288: Are these seeds used by other species? This may contribute to the co occurence of wild boar and other seed eater species and complicate the picture you are analysing.

**Response:** There are many mammals and some birds, such as those from the Psittacidae and Corvidae families, that extensively consume this resource. Araucaria seeds are consumed by peccaries and especially by rodents, including smaller ones (which probably attract carnivores). Thus, indeed other seed eaters could blur our picture regarding the effects of wild boar. However, these seeds are widespread across the Conservation Unit. As a result, we could not evaluate the effects of possible differences throughout the study area.

Line 291: root

**Response:** We have applied this change.

Line 298: As said in previous commets you have difficuties to determine if this is the outcome of lack of influence of wild boar or of environmental characteristics that can buffer detrimental effects thanks to e.g. food availability. You definitely need to assess environmental conditions in yours samplig spots

**Response:** We understand the reviewer’s concern. But However, this addresses a different question, which involves other difficulties. As explained in our responses to several previous comments, this Conservation Unit is relatively small and many mammals are very mobile. We believe that our sampling points were enough to cover the Conservation Unit and record most mammals. However, it is crucial to understand that the central question of our study was not to evaluate spatial correlations, but temporal correlations. We compared the activity patterns of mammals when wild boars were present (active at each sample site) versus the activity patterns when they were not present (using the proximities) at that sample site.

Line 308: You can not know this because you have no idea of the variations you may have found in species composition and abundance if the wild boar was not present .

**Response:** Indeed we cannot confirm this. We do not have a spatial absence of wild boar for comparison. This section was deleted.

Line 316: I totally agree with the hypotheses give in the last two sentences but it would be useful to add some data supporting them.

**Response:** Unfortunately, reviewing this specific topic illuminated doubts regarding a bigger abundance of rodents in the Araucaria forest when compared to other environments. Therefore, we discarded the last section of this paragraph.

Line 332: This make no sense as wild boar is an obvious prey species for pumas. Do you have any ideas on the diet of pumas'?

**Response:** As indicated in response to a previous comment, wild boar are not necessarily always prey for puma. The diet of puma is known for its plasticity. In a revision of this topic in South America we found information about the consumption of wild boar by pumas, which was associated with a reduction of natural prey species, especially in arid environments (Andes region). There is no information about the consumption of wild boar in forested areas, although obviously this can occur. However, forested habits tend to present a more diverse prey base. Even in study areas with predation of wild boar by pumas, the consumption of this prey is usually not frequent (with a frequency of less than 10% of the samples). Finally, considering a latency of almost 9 days between records of wild boar and puma, we are convinced that pumas are not actively foraging on this species. Nevertheless, we included information regarding the revision of the puma diet and some comments about the latency period between records of wild boar and puma.
